# Elevated serum apelin levels in breast cancer patients: A potential biomarker with limited histopathological correlations

**DOI:** 10.3389/fonc.2025.1513693

**Published:** 2025-09-22

**Authors:** Adel Soltanizadeh, Aliasghar Tirgar, Yasaman Zamanian, Mehran Ilaghi, Sarah Aflatoonian, Ali Karamoozian, Mohammad Shabani, Elham Jafari, Ali Mahmoudabadi, Vahid Moazed

**Affiliations:** ^1^ Department of Medical Oncology, Faculty of Medicine, Kerman University of Medical Sciences, Kerman, Iran; ^2^ Department of Medical Education, Education Development Center, Kerman University of Medical Sciences, Kerman, Iran; ^3^ Student Research Committee, Kerman University of Medical Sciences, Kerman, Iran; ^4^ Institute of Neuropharmacology, Kerman Neuroscience Research Center, Kerman University of Medical Sciences, Kerman, Iran; ^5^ Department of Radiation Oncology, Kerman University of Medical Sciences, Kerman, Iran; ^6^ Department of Biostatistics and Epidemiology, Kerman University of Medical Sciences, Kerman, Iran; ^7^ Modeling in Health Research Center, Institute for Futures Studies in Health, Kerman University of Medical Sciences, Kerman, Iran; ^8^ Department of Pathology, Pathology and Stem Cell Research Center, School of Medicine, Kerman University of Medical Sciences, Kerman, Iran; ^9^ Department of Medical Genetics, Kerman University of Medical Sciences, Kerman, Iran

**Keywords:** apelin, breast cancer, clinicopathological parameters, serum marker, histopathological

## Abstract

**Background:**

Apelin, a peptide implicated in various physiological processes, has been shown to be involved in cancer development and progression. However, its role in breast cancer remains unclear. This study aimed to investigate serum apelin levels in breast cancer patients and explore potential associations with clinicopathological features.

**Methods:**

This study involved 137 histopathologically-confirmed female breast cancer patients and 71 healthy controls. Serum apelin levels were measured using enzyme-linked immunosorbent assay (ELISA) and patients’ clinicopathological data was collected retrospectively. Serum apelin levels were compared between the patients and control groups. Moreover, Youden’s J index and the receiver operating characteristic (ROC) curve analysis were utilized to select the optimal cut-off point to differentiate patients and healthy controls. A generalized linear model (GLM) was used to investigate the association of each histopathological variable with serum apelin levels.

**Results:**

Serum apelin levels were significantly higher in breast cancer patients (343.61 ± 182.69 pg/mL) compared to healthy controls (67.37 ± 30.18 pg/mL, p<0.001). ROC curve analysis revealed excellent discriminative ability of serum apelin in distinguishing breast cancer patients from healthy controls (AUC = 0.94, 95% CI: 0.91-0.98). The optimal cut-off value for serum apelin was determined to be 122.48 pg/mL, yielding 89% sensitivity and 97% specificity. However, GLM analysis found no statistically significant associations between serum apelin levels and clinicopathological features, including age, tumor size, histologic grade, lymphovascular invasion, lymph node involvement, microcalcification, *in situ* components, necrosis, Ki67 expression, molecular subtypes, and clinical stage.

**Conclusions:**

Our findings suggest a potential role for serum apelin in breast cancer pathogenesis or progression. The high discriminative ability of serum apelin indicates its promise as a biomarker for breast cancer detection. However, the lack of association between serum apelin levels and specific clinicopathological features suggests a limited prognostic value and a complex role in breast cancer biology that warrants further investigation.

## 1 Introduction

In 2020, for the first time, female breast cancer emerged as the most prevalent diagnosed malignancy worldwide with an estimated 2.26 million new cases reported (1). Projections for the year 2040 indicate an escalation in the global burden of breast cancer, with an expected annual increase to over 3 million new cases (2). On the other hand, the latest global statistics reveal that breast cancer stood as the predominant cause of cancer-related mortality among women, ranking fifth in overall cancer deaths (3). Based on all this alarming evidence, breast cancer continues to constitute a significant global health concern, notwithstanding the rapid advancements within this domain.

The shortage of sensitive markers for early diagnosis and monitoring of breast cancer progression presents a notable challenge in the provision of effective treatment, resulting in suboptimal outcomes and decreased survival. Identifying objective and trustworthy biomarkers to improve cancer screening and treatment is essential, which is vital for advancing personalized and precision medicine. Initiatives focused on uncovering novel proteins and various molecules implicated in the development and progression of breast cancer have demonstrated promising findings (4–6).

One of these biomolecules, with an approved effect on breast cancer pathology, is apelin, which has received attention in the scientific community since 1998 (7, 8). This protein, found at elevated levels in the serum of patients diagnosed with breast cancer (6), is an endogenous ligand for the G-protein coupled receptor known as Apelin receptor (APJ) and was initially extracted from bovine stomach tissue (9). Subsequent research has revealed apelin expression in heart muscles, brain, kidneys, liver, lungs, spleen, mammary glands, placenta, and gastric mucosa (10). This small peptide plays a crucial role in numerous essential physiological functions, including angiogenesis, cell migration, cellular permeability, energy metabolism, neuroendocrine regulation, fluid homeostasis, and glucose metabolism (11, 12). It is particularly recognized for its impact on the cardiovascular system, exhibiting both inotropic effects and functioning as a vasopressor and vasodilator (13).

Recent investigations have demonstrated that the APJ is significantly overexpressed in tumor tissues, especially in those that have undergone metastasis (14–16). On the other side, increased levels of apelin expression have been observed in several cancer forms, particularly in breast cancer, where such elevations correlate with diminished survival durations and a higher incidence of cancer recurrence (17–19). Actually, apelin induces the maturation of tumor blood capillaries and prompts tumor vascularization (20). Additionally, it exhibits lymphangiogenic properties that influence tumor progression and lymph node metastasis, thereby mediating cancerous cells’ survival, proliferation, invasion, and metastasis (8, 11, 13, 21). It exerts the mentioned effects by stimulating the proliferation of lymphatic endothelial cells through the engagement of the ERK, STAT3, and PI3K signaling pathways (14, 21).

Research into tissue apelin levels has been comprehensive, revealing some discrepancies in findings while suggesting a link to clinicopathological traits. Since tumor tissue samples are often difficult to obtain, serum samples provide a less invasive method that may prove more effective for measuring apelin concentrations in various situations, including advanced cancers (17, 18, 22–24). Also, for clinical use, serum apelin holds the potential for enhancing cancer screening and providing personalized care for patients. In contrast, measures of tissue apelin are often only applicable after identifying the tumor (6). Thus, it is crucial to determine the optimal cutoff points of serum apelin concentration that indicate the presence of disease and its related processes. This can help identify breast cancer patients earlier, improving health outcomes and overall survival.

On the other side, breast cancer is a complex and heterogeneous disease, and its prognosis may vary depending on the combined characteristics of the tumor and clinicopathologic features (25). Hence, investigating the correlation between apelin and clinicopathologic characteristics is imperative in evaluating whether these relations possess sufficient robustness to anticipate outcomes such as the probability of lymphatic system involvement as a primary sign of metastasis and to figure out the specific associations that represent shorter survival or worse prognoses.

The aims of the present study included investigating the correlations of apelin in breast cancer patients with different clinicopathological features. These features comprise age, tumor size, histologic grade, lymphovascular invasion, lymph node involvement, microcalcification, *in situ* components, necrosis, Ki67 expression, and molecular subtypes. The study also aimed to determine the optimal cut-off value for serum apelin that indicates the presence of the disease.

## 2 Methods

### 2.1 Study design and participants

This study comprised female breast cancer patients who were histopathologically confirmed as invasive ductal carcinoma by core needle biopsy with immunohistochemistry staining. The participants were referred to the outpatient clinic of Kerman University of Medical Sciences, Kerman, Iran, between March 2021 and June 2023. We excluded individuals with a past medical history of other malignancies and any other inflammatory and severe metabolic diseases. We also recruited healthy individuals (in an approximately 1:2 ratio relative to patients) as controls from clinic attendants for routine check-ups. These individuals were not diagnosed with any prior malignancies and metabolic disease and were sex- and aged-matched as the breast cancer patients. All patients and control members provided written informed consent before proceeding with the study. This study was conducted under the approval of the Ethics Committee of Kerman University of Medical Sciences (Ethics code: IR.KMU.AH.REC.1402.080).

### 2.2 Serum sampling

We took 5cc blood samples from the peripheral venous vessels of breast cancer patients and control-group participants, which were then kept in tubes containing clot activators allowed to clot at room temperature for 10–15 min before centrifugation. We prepared serum samples by centrifuging the clotted whole blood for 10 min at 2000 RPM. The serum was then transferred by sampler to sterile cryotubes and stored in a -60°C refrigerator until analysis.

### 2.3 Enzyme-linked immunosorbent assay

Apelin levels in human serum were measured using enzyme-linked immunosorbent assay (ELISA) methodology based on the biotin double-antibody sandwich technique. The assay followed the manufacturer’s instructions (*Human Apelin (APLN) ELISA Kit*, *ZellBio GmbH, Ulm, Germany*). The sensitivity of the apelin assay was 2.63 pg/mL. The inter and intra-assay coefficients of variation were <12% and <10%, respectively. To perform the assay, an antibody specific to apelin was pre-coated onto a 96-well plate. A competitive inhibition reaction was initiated between biotin-labeled and unlabeled apelin with the pre-coated antibody specific to apelin. The unbound conjugates were washed away, and Streptavidin-HRP conjugated to horseradish peroxidase was added to each microplate well and incubated. Then, the substrate solution was added, and the color changed to yellowish, appearing only in wells containing apelin by adding the acidic stop solution. The Optical Density (O.D) value was measured spectrophotometrically at 450 nm wavelength in a microplate reader Synergy™ Multi-purpose Microplate Reader (*Nano Mabna; Iran*) equipped. Apelin concentration was calculated based on the standard curve by Gen5 Software (*BioTek; the USA*).

### 2.4 Pathological assessments

Pathological data from breast cancer patients were collected retrospectively. The pathological assessment in our center involved breast mass sampling through core-needle biopsy (CNB) guided by ultrasound. A little incision (approximately ¼ inch) was occasionally made in the breast, allowing the insertion of a biopsy needle to extract tissue samples. In CNB, a thin needle was used (following local anesthesia) within the biopsy site, with ultrasound guidance ensuring the right spot. Afterward, tissue samples were fixed in 50% ethanol for 4–12 hours, then in 10% neutral buffered formalin for 6 hours. After formalin fixation, immunohistochemistry (IHC) was carried out using monoclonal mouse antibodies for estrogen receptor (*ER, xbio, Framingham, Massachusett, USA*), progesterone receptor (*PR, xbio, Framingham, Massachusett, USA*), and herceptin (*xbio, Framingham, Massachusett, USA*). Tissue sections were deparaffinized, rehydrated, and exposed to heat-induced epitope retrieval in citrate buffer (pH 6.0) using an electric pressure cooker at 12–15 PSI and 120°C for 3 minutes, followed by a 10-minute cooling period before immunostaining. All slides underwent hydrogen peroxide treatment for 5 minutes and incubation with primary antibodies for 30 minutes at room temperature, with TBS washes between incubations.

Herceptin staining involved immersion and incubation of slides in 10 mmol/L citrate buffer in a water bath at 95–99 °C for 40 minutes. Post-decanting, sections were washed and pre-soaked before staining, then treated with peroxidase blocker, incubated with anti-HER2 protein or negative control, stained with chromogen substrate (DAB), counterstained with hematoxylin, and coverslipped. Finally, the pathologist’s evaluation of the samples included scoring and grading based on ER, PR, and HER2 staining patterns and intensity.

In the process of Ki67 staining for immunohistochemistry, tissue samples were fixed in 10% neutral buffered formalin for 6 to 10 hours, followed by embedding in paraffin. After the deparaffinization and rehydration, antigen retrieval was performed using citrate buffer. The samples were stained with a monoclonal antibody against Ki-67 (*clone MIB-1,xbio, Framingham, Massachusett, USA*) and incubated accordingly. Finally, the samples were counterstained with hematoxylin and coverslipped for examination. A 14% cut-off value was used to categorize Ki-67 expression as low and high expression (26).

Tumor staging and other histopathological features were revised by the same expert pathologist using the 8^th^ version of the Union for International Cancer Control (UICC) TNM classification of malignant tumors. Clinical staging was performed according to the classification by American College of Surgeons and American Cancer Society.

### 2.5 Statistical analysis

Statistical analysis was performed using SPSS version 22 *(IBM, SPSS Inc., USA)* and *R 4.1.1* software. An independent sample t-test was employed to compare the serum apelin level between the patients and control group after checking and verifying the normality and homogeneity of variances. To select the optimal cut-off point with the highest sensitivity and specificity to differentiate patients and healthy controls, the Youden’s J index was employed through the *“cutpointr”* package in R software. Additionally, in order to evaluate the model’s performance, the receiver operating characteristic (ROC) curve was plotted, and the area under the curve (AUC) was calculated. To investigate the association of each histopathological variable with serum apelin levels, considering the structure of the study data, a generalized linear model (GLM) was utilized based on the Inverse Gaussian distribution for the response variable (serum apelin level). The significance level was set at 0.05 for all statistical tests.

## 3 Results

Data from a total of 137 breast cancer patients were investigated in this study. The mean (± SD) age of the patients was 49.48 (± 10.45) years. The majority of patients had a tumor histologic grade II (84.6%), followed by grades III (26.9%) and I (8.5%). The most prevalent molecular subtypes were Luminal B-like (HER2 negative), Luminal B-like (HER2 positive), and Basal-like (Triple Negative) in 43.5%, 20.6%, and 13.0% of cases, respectively. The majority of patients had a clinical stage of IIA (34.8%) and IIB (25.0%). High Ki67 expression was reported in 78.4% of the patients. The detailed clinicopathological characteristics of the patients are demonstrated in **Table 1**.

**Table 1:** Demographic and clinicopathological characteristics of breast cancer patients.

Characteristics		N	%
Age	<55 years	95	69.9
	≥55 years	41	30.1
Tumor Localization	Right	65	48.1
	Left	70	51.9
Tumor Size	<2 cm	18	19.6
	≥2cm	74	80.4
Tumor Histologic Grade	I	11	8.5
	II	84	64.6
	III	35	26.9
Perineural Invasion	Present	16	57.1
	Not observed	12	42.9
Lymphovascular Invasion	Present	88	81.5
	Not observed	20	18.5
Microcalcification	Present	71	68.9
	Not observed	32	31.1
*In situ* components	Present	89	80.9
	Not observed	21	19.1
Necrosis	Present	61	61.6
	Not observed	38	38.4
Lymphocytic Host Response	Not observed	5	6.8
	A	18	24.7
	B	49	67.1
	C	1	1.4
Postoperative lymph node involvement	Free	57	53.8
	Involved	49	46.2
Ki67 Expression	High	98	78.4
	Low	27	21.6
Molecular Subtype	Luminal A-like	14	10.7
	Luminal B-like (HER2 negative)	57	43.5
	Luminal B-like (HER2 positive)	27	20.6
	HER2-enriched	16	12.2
	Basal-like (Triple Negative)	17	13.0
Clinical Stage	IA	24	18.2
	IB	8	6.1
	IIA	46	34.8
	IIB	33	25.0
	IIIA	18	13.6
	IIIB	1	0.8
	IIIC	2	1.5

Our analysis revealed a significant difference in serum apelin levels between breast cancer patients and healthy controls. The mean (± SD) serum apelin level in breast cancer patients was 343.61 (± 182.69) pg/mL, which was significantly higher than that of healthy controls at 67.37 (± 30.18) pg/mL (p<0.001) (**Table 2**).

**Table 2:** Comparison of serum apelin level in breast cancer patients and healthy controls.

Group	Serum apelin level (pg/mL) mean ± SD	P-value
Breast Cancer	343.61 ± 182.69	<0.001
Healthy Control	67.37 ± 30.18	

SD: Standard Deviation

**Figure 1:**
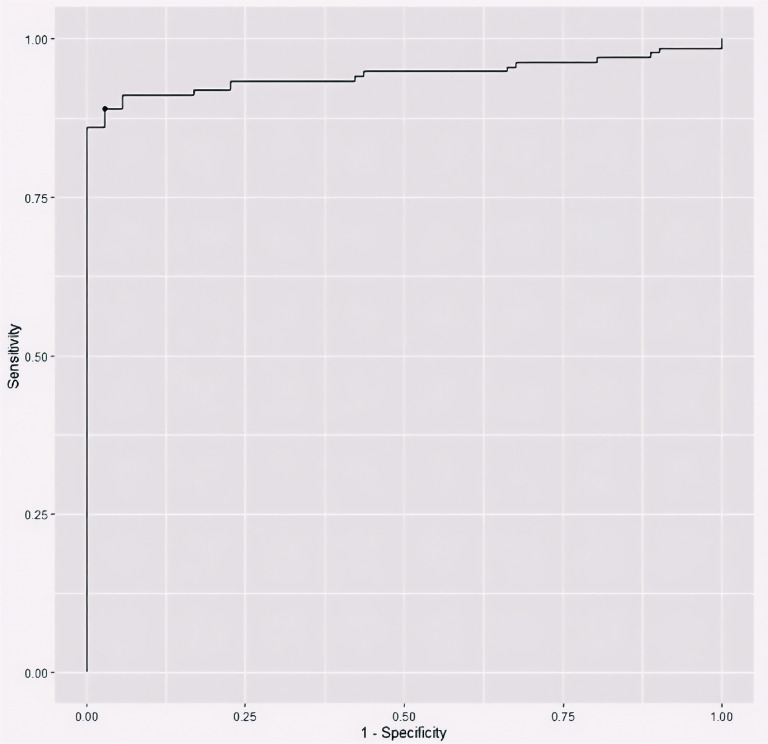
The receiver operating characteristic (ROC) curve plotting the discriminative utility of serum apelin level between breast cancer patients and healthy controls.

To evaluate the utility of serum apelin as a potential biomarker for breast cancer, we performed a ROC curve analysis (**Figure 1**). The area under the ROC curve (AUC) was 0.94 (95% CI: 0.91-0.98), indicating excellent discriminative ability between breast cancer patients and healthy individuals. Using Youden’s J index, we determined the optimal cut-off value of 122.48 pg/mL for serum apelin level to differentiate between breast cancer patients and healthy controls, which yielded a sensitivity of 0.89 (95% C.I: 0.84-0.94) and a specificity of (95% C.I: 0.94-1.00) (**Table 3** and **Figure 1**).

**Table 3:** Youden’s J index and ROC curve criteria for serum apelin level utility in differentiating between breast cancer patients and healthy controls.

Optimal apelin cut point	122.48
Youden’s J	0.86
Accuracy	0.92
Sensitivity	0.89 (95% C.I: 0.84-0.94)
Specificity	0.97 (95% C.I: 0.94-1.00)
Area under the ROC curve (AUC)	0.94 (95% C.I: 0.91-0.98)
Standard error for AUC	0.02

The GLM analysis did not reveal any statistically significant associations between serum apelin levels and the clinicopathological variables examined, including age, tumor size, histologic grade, lymphovascular invasion, lymph node involvement, microcalcification, *in situ* components, necrosis, Ki67 expression, molecular subtypes, and clinical stage (all p-values > 0.05) (**Table 4**).

**Table 4:** Generalized linear model based on inverse Gaussian distribution exploring the clinicopathological predictive factors of serum apelin level in breast cancer patients.

Variables		β	95% CI for β	P-value
Age	<55	0.14	(-19.17, 18.89)	0.98
	>55	0.00*		
Tumor Size	<2	6.11	(-41.65, 29.43)	0.74
	>2	0.00*		
Tumor Histologic Grade	I	-12.82	(-39.85, 14.09)	0.35
	II	-1.65	(-24.61, 21.30)	0.88
	III	0.00*		
Lymphovascular Invasion	Present	3.45	(-21.04, 27.95)	0.78
	Not seen	0.00*		
Postoperative Lymph Node Involvement	Involved	-1.38	(-23.69, 20.92)	0.90
	Free	0.00*		
Microcalcification	Present	-0.84	(-25.28, 23.61)	0.94
	Not seen	0.00*		
*In situ* Component	Present	-2.80	(-31.69, 26.09)	0.85
	Not seen	0.00*		
Necrosis	Present	6.25	(-16.04, 28.54)	0.58
	Not seen	0.00*		
Ki67	Low expression	6.48	(-33.25, 20.29)	0.63
	High expression	0.00*		
Molecular Subtype	Luminal A-like	5.92	(-46.46, 58.30)	0.82
	Luminal B-like (HER2 negative)	-9.57	(-42.57, 23.43)	0.57
	Luminal B-like (HER2 positive)	-9.59	(-44.62, 25.44)	0.59
	HER2-enriched	1.24	(-44.45, 46.93)	0.96
	Basal-like (Triple negative)	0.00*		
Clinical stage	IA	4.58	(-28.46, 37.62)	0.78
	IB	15.36	(-53.09, 83.83)	0.66
	IIA	1.87	(-24.21, 27.95)	0.88
	IIB	1.08	(-26.59, 28.75)	0.93
	III	0.00*		

β, Regression coefficient; CI, Confidence interval, * Reference category

## 4 Discussion

This study investigated serum apelin levels in breast cancer patients and their potential association with various clinicopathological features. Overall, we observed significantly elevated serum apelin levels in breast cancer patients compared to healthy controls, but no associations with clinicopathological features of the patients.

Several previous studies have reported altered tumoral apelin expression in breast cancer patients. In a study by Hu et al., the protein and mRNA expression of apelin was higher in breast cancer tissues compared to normal breast tissue (27). However, another study by Baran and colleagues showed that apelin expression was lower in the breast tissue of patients with invasive breast carcinoma compared to the normal breast tissue (28). On the other hand, studies on the serum apelin level in breast cancer patients have been limited. A recent systematic review of studies assessing serum or plasma apelin levels in all cancer types in the past decade only detected two studies assessing the circulating apelin levels in breast cancer patients (6), from which only in one study, the serum apelin level was compared with a control group, showing that the serum apelin levels were higher in early-stage postmenopausal breast cancer patients compared to healthy controls (29). The marked difference in serum apelin levels between patients and controls in our study suggests that apelin may play a role in breast cancer pathogenesis or progression. Furthermore, the ROC curve analysis in this study demonstrated excellent discriminative ability of serum apelin in distinguishing breast cancer patients from healthy controls, with an AUC of 0.94. This high AUC value, coupled with the optimal cut-off point of 122.48 (yielding 89% sensitivity and 97% specificity), suggests that serum apelin could potentially serve as a valuable biomarker for breast cancer detection.

To better contextualize our findings, it is important to note that previous experimental studies have identified several mechanisms through which apelin may influence cancer development and progression. So far, several mechanisms have been proposed for the potential role of apelin in cancer, including its involvement in angiogenesis, cell proliferation, and metastasis (19, 30). Studies have shown that apelin can activate the PI3K/Akt and MAPK/ERK signaling pathways, which are crucial for cell survival, growth, and migration (31, 32). Accordingly, it has been suggested that apelin contributes to tumor growth, angiogenesis, and metastasis (30, 33). Apelin has been shown to stimulate angiogenesis by promoting the proliferation and migration of endothelial cells (34), which could support tumor vascularization and growth. Recent findings have also shown that blocking the apelin/APJ axis could inhibit tumor growth and that APJ antagonists can also boost dendritic cell vaccine efficacy in controlling apoptotic, angiogenic, and metastatic properties of breast tumors (35). Our current clinical findings of elevated serum apelin in breast cancer patients are consistent with these previously reported biological activities, although the precise mechanisms underlying the elevation in our patient cohort remain to be elucidated in future studies.

Importantly, our analysis did not reveal any significant associations between serum apelin levels and the clinicopathological features examined, including tumor size, grade, lymphovascular invasion, lymph node involvement, molecular subtypes, and clinical stage. Similar to our findings, in a study by Grupińska et al., baseline apelin serum levels (1.30 ± 0.40 ng/mL) had no correlations with tumor size, histopathological grade, and regional lymph node metastasis of breast cancer patients (7). However, apelin level was associated with positive HER2/neu status (7). Nevertheless, this lack of association between serum apelin level and clinicopathological features contrasts with some previous studies that have reported correlations between tumoral apelin expression and certain prognostic factors in breast cancer. In a study by Hu and colleagues, apelin expression in breast cancer tissue had significant correlations with tumor size, stage, histological type, microvessel density, lymphatic vessel density, and lymph node metastasis (27). The findings on increased lymphatic vessel density and lymph node metastasis suggest that apelin may play an essential role in lymphangiogenesis as a route for the metastasis of cancer. Moreover, higher tumoral apelin expression has been associated with both worse disease-free survival and overall survival (27), indicating its potential as a prognostic factor. Interestingly, high tumoral apelin expression has been found to be associated with reduced response to neoadjuvant chemotherapy in breast cancer patients (36).

Overall, the absence of significant associations with clinicopathological features suggests that while serum apelin levels are elevated in breast cancer patients, they may not be prognostic factors indicative of specific tumor characteristics or disease severity. This study has several strengths, including a relatively large sample size of breast cancer patients and a comprehensive analysis of potential clinicopathological features. However, several limitations must be noted. The cross-sectional nature of the study prevents us from drawing conclusions about the causal relationship between apelin and breast cancer development or progression. Moreover, while the discriminative performance of serum apelin for present cancer is promising, our study design does not allow us to make definitive claims about apelin’s performance specifically in early-stage disease detection. Future prospective studies with stage-stratified analysis and longitudinal follow-up of at-risk individuals would be necessary to determine if apelin elevations can be detected at the earliest, most treatable stages of breast cancer, and whether screening for elevated apelin levels could improve patient outcomes. The high sensitivity and specificity observed in our study provide a strong rationale for such investigations. Additionally, we did not assess apelin expression in tumor tissues, which could provide complementary information to serum levels. We encourage research directions to include longitudinal studies to evaluate changes in serum apelin levels during cancer progression and treatment, as well as mechanistic studies to elucidate the specific roles of apelin in breast cancer biology. Moreover, investigating the potential of apelin as a therapeutic target in breast cancer could be a promising avenue for future research.

## 5 Conclusions

In conclusion, our study demonstrates significantly elevated serum apelin levels in breast cancer patients, with high discriminative ability between patients and healthy controls. While serum apelin shows promise as a potential biomarker for breast cancer detection, its lack of association with specific clinicopathological features suggests a limited prognostic role and a complex role in breast cancer biology that warrants further investigation.

## Data Availability

The original contributions presented in the study are included in the article/supplementary material. Further inquiries can be directed to the corresponding author.
